# Multifactorial Activation of NLRP3 Inflammasome: Relevance for a Precision Approach to Atherosclerotic Cardiovascular Risk and Disease

**DOI:** 10.3390/ijms21124459

**Published:** 2020-06-23

**Authors:** Andrea Baragetti, Alberico Luigi Catapano, Paolo Magni

**Affiliations:** 1 Dipartimento di Scienze Farmacologiche e Biomolecolari, Università degli Studi di Milano, 20133 Milan, Italy; andrea.baragetti@unimi.it (A.B.); alberico.catapano@unimi.it (A.L.C.); 2 SISA, Center for the Study of Atherosclerosis, Bassini Hospital, 20092 Cinisello Balsamo, Italy; 3 IRCCS Multimedica Hospital, 20099 Milan, Italy

**Keywords:** cardiovascular disease, atherosclerosis, NLRP3 inflammasome, low-grade inflammation, interleukin-1β, single nucleotide polymorphism

## Abstract

Chronic low-grade inflammation, through the specific activation of the NACHT leucine-rich repeat- and PYD-containing (NLRP)3 inflammasome-interleukin (IL)-1β pathway, is an important contributor to the development of atherosclerotic cardiovascular disease (ASCVD), being triggered by intracellular cholesterol accumulation within cells. Within this pathological context, this complex pathway is activated by a number of factors, such as unhealthy nutrition, altered gut and oral microbiota, and elevated cholesterol itself. Moreover, evidence from autoinflammatory diseases, like psoriasis and others, which are also associated with higher cardiovascular disease (CVD) risk, suggests that variants of NLRP3 pathway-related genes (like NLRP3 itself, caspase recruitment domain-containing protein (CARD)8, caspase-1 and IL-1β) may carry gain-of-function mutations leading, in some individuals, to a constitutive pro-inflammatory pattern. Indeed, some reports have recently associated the presence of specific single nucleotide polymorphisms (SNPs) on such genes with greater ASCVD prevalence. Based on these observations, a potential effective strategy in this context may be the identification of carriers of these NLRP3-related SNPs, to generate a genomic score, potentially useful for a better CVD risk prediction, and, possibly, for personalized therapeutic approaches targeted to the NLRP3-IL-1β pathway.

## 1. Chronic Low-Grade Inflammation and Atherosclerotic Cardiovascular Risk and Disease: Moving Above and Beyond the Lipid Profile?

The risk of developing atherosclerotic cardiovascular disease (ASCVD) is well-known to result from the individual combination of genetic factors and environmental factors (exposome), the former being classically defined as “non-modifiable” and the latter as “modifiable” [1]. Recent evidence suggests a lesser clear-cut partitioning, since, for example, epigenetic changes may permanently link the two clusters of factors [2]. Such pathogenetic complexity bears important consequences on the practical and clinical side for most non-communicable diseases and, specifically, for ASCVD, although it may represent an opportunity for precision medicine approaches.

Elevated circulating cholesterol is an established and undisputable cardiovascular (CV) risk factor [3], and the evaluation of circulating low-density lipoprotein (LDL-C) level and its management when abnormally elevated is the main focus of most guidelines in this field [4]. Moreover, validated CV risk calculators, including the Systemic Coronary Risk Evaluation (SCORE) [5] and the Framingham Risk Score (FRS) [6], are indeed based upon lipid biomarkers, in addition to other relevant clinical indicators. The clinical availability of the potent cholesterol synthesis inhibitors, statins, clearly changed the cardiovascular disease (CVD) scenario [7], but, due to the extensive trials conducted with these drugs, the important concept of residual CV risk has been highlighted as another important area of experimental and clinical work [8].

Although higher LDL-C levels are causally related to atherosclerotic CVD risk and incidence, and LDL-C lowering is associated with the proportional reduction of CVDs, a relevant share of CV risk thus appears to be related to additional lipid-independent factors that need to be addressed by research and potentially targeted in clinics. Among these pathogenic mechanisms, the focal and systemic inflammation hypothesis of ASCVD has been put forward for a long time and has been strongly corroborated by a large set of experimental and clinical studies [1], including recent pivotal trials, leading to convincing experimental and clinical evidence [9]. Thus, it seems scientifically sound to move beyond and above cholesterol in the management of ASCVD risk, obviously not neglecting the current evidence on the high-intensity management of lipid markers [4], but extending the individual assessment to environmental, genetic and epigenetic components involved in the modulation of low-grade chronic inflammation.

In this regard, current evidence indeed indicates that ASCVD pathophysiology appears to involve the activation of a selected pro-inflammatory pathway, the NACHT (named after NAIP, CIITA, HET-E, and TP1) leucine rich-repeat and PYD-containing (NLRP)3 inflammasome interleukin-1β (IL-1β) pathway [9], but, possibly, not of other pro-inflammatory mechanisms [9,10,11]. 

The elucidation of such individual ASCVD risk profile may then enable us to implement specific and effective prevention and therapeutic strategies, tailored according to precision medicine criteria.

Based on these observations, the purpose of this review is:

1. To discuss the role of different factors activating the NLRP3 inflammasome-IL-1β pathway in the progression of ASCVD, highlighting the related molecular mechanisms leading to low-grade inflammation;
2. To report the current evidence about the individual impact of such pro-inflammatory mechanisms on the personal ASCVD risk, in the context of a precision medicine approach to CVD risk assessment and prevention, based on genomic analysis.

## 2. NLRP3 Inflammasome Structure and Function

### 2.1. NLRP3 Inflammasome Functions

**Figure 1:**
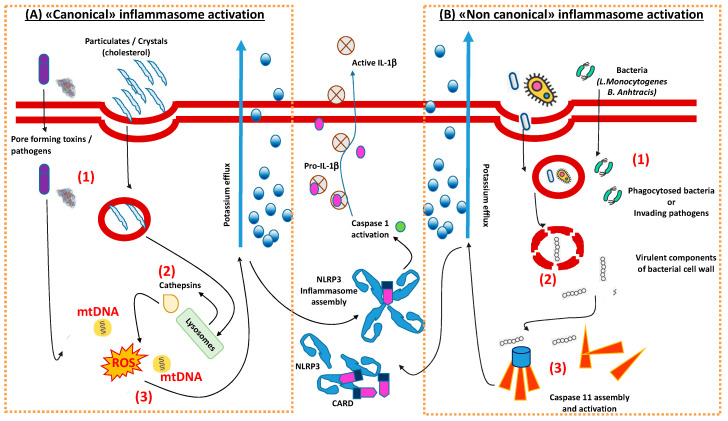
Summary of cellular mechanisms leading to the activation of the NLRP3 inflammasome. Depending on the pathogenic activator, two different types of cellular mechanisms lead to the assembly and activation of NLRP3 inflammasome, crucial for the production of atherogenic molecules (interleukin (IL)-1β). (**A**) The “canonical pathway” is (1) activated by the phagocytosis of molecular products (e.g., cholesterol crystals in the atherosclerotic lesion) or peculiar pathogen-associated molecules or cellular stimuli (e.g., ATP via the P2X7 receptor), and involves (2) the activation of lysosomial cathepsins and (3) pro-apoptotic mitochondrial signals. (**B**) The “non-canonical pathway” evolutionary developed for the phagocytosis of peculiar bacterial species, (1) either by endocytosis or by direct invasion; this in turn (2) activates programmed digestion of these products with the release of virulent proteins from the wall membrane of bacteria which (3) induce the assembly of caspase 11. Both mechanisms induce massive efflux of potassium, which is then crucial for the assembly of the components of the NLRP3 inflammasome. This will finally turn pro-caspase 1 into active caspase 1 to favor the release of the final mature form of IL-1β. mtDNA, mitochondrial DNA; ROS, reactive oxygen species; CARD, caspase activation and recruitment domain.

(Acute) inflammation is usually triggered in response to infections and/or tissue damages, representing a protective process, which includes the recruitment of immune cells that coordinate pathogen clearance and tissue repair at the affected site. Besides the adaptive immune arm, which consists of boosted antigen-presenting cells (APC) supporting the pivotal activity of lymphocytes to recognize an exceptional variety of antigens changing over time, innate immune has maintained a complex system of antigen-recognition across evolution. This system is conceived to guarantee an immediate response against a fixed number of pathogens with a multitude of germline-encoded “pattern recognition receptors” (PRRs) [12]. As the plethora of pathogens ranges from bacterial lipids to nucleic acids (PAMPs), secretion systems or dead cells-residues (DAMPs) from pyroptosis, innate immune cells developed the differentiation of an entire class of receptors, that can be divided into two major branches: (1) transmembrane receptors, such as the Toll-like receptors (TLRs) and the C-type lectin receptors (CLRs); and (2) intracellular receptors (such as the RIG-I-like receptors, the AIM-2 like receptors and the nucleotide-binding domain and leucine-rich repeat-containing (NLR) proteins) [12]. From a functional point of view, while the activation of some of these receptors involves the switching of downstream intracellular and intra-nuclear pathways (the nuclear factor-kB (NF-kB), activator protein 1 (AP1), and interferon regulatory factors (IRFs)), others involve the aggregation of multiple proteins complexes, the “inflammasomes”, leading to the conversion of pro-proteins and zymogens into pro-inflammatory proteins, secreted for extracellular and/or paracrine activities [12,13,14]. Among the second group, from a structural point of view, two types of inflammasomes can be recognized: 1) “canonical inflammasomes”, turning pro-caspase 1 into caspase 1, (the “NLRs” family and the ALR AIM2 type); and 2) “non-canonical inflammasomes”, turning pro-caspase 11 into caspase 11 [15] (Figure 1). The activation of caspase 1 or caspase 11 depends on the type of pathogen. Both caspase 1 and 11 trigger pyroptosis, but only caspase-1 processes IL-1β and IL-18. IL-1βis a pyrogenic cytokine that also promotes adaptive T helper 1 (Th1), Th17, and humoral immunity [12]. In particular, canonical inflammasomes have been deeply characterized from structural and functional points of view; moreover, their respective ligands have been identified, thereby having constructed the whole spectrum of antigens against which this immune complex is conceived. In particular, while DNA viruses and *Listeria Monocytogenes* and *Bacillus Anthracis* have been identified as principal antigenic ligands for AIM2 and for NLRP1 respectively, the NLRP3 inflammasome complex seems to broadly interact with endogenous, intracellular mediators, thereby representing a crucial inflammasome complex between cellular pathology, metabolism and inflammation [15].

### 2.2. Molecular Structure of NLRP3 Inflammasome

NLRP3 mainly consists of: (1) a cytosolic sensor molecule NLRP3; (2) an adaptor protein apoptosis-associated speck-like protein containing a caspase activating recruitment domain (ASC); (3) a cysteine protease pro-caspase-1 as the effector molecule; (4) a C-terminal leucine-rich repeat (LRR) domain which recognizes PAMPs and ligands; and (5) a conserved central nucleotide binding and oligomerization domain (NOD or NACHT) with ATPase activity essential for self-oligomerization [16]. The ASC interacts with the cytosolic sensor NLRP3 through its N-terminal PYD domain [17], which binds pro-caspase-1 via its caspase activation and recruitment domain (CARD) (C-terminal domain) [18]. Pro-caspase-1 recruitment, which is mediated by ASC, contributes to caspase-1 oligomerization and auto-proteolytic conversion of the pro-enzyme into its active form [19], eliciting maturation and secretion of mature IL-1β and IL-18 maturation and secretion. Therefore, caspase-1 activation from its precursor is the crucial trigger of the NLPR3 inflammasome activation, which is second to the recognition of multiple ligands. In fact, besides bacterial- and fungal-derived products, NLRP3 is also activated by pore-forming toxins, crystals and aggregates like β-amyloid and DAMPs like ATP hyaluronan. NLRP3 assembly and activation is ensured by the activation of the IL1 Receptor (IL1R) systems, which are structurally a proxy of the TLRs. The mechanisms by which NLRP3 acts as a cellular monitor of the invasion of all these antigens are therefore complex and derive from intense investigation.

## 3. NLRP3 Inflammasome Activation by Common Ligands

It can be essentially assumed that NLRP3 may be activated through: (1) interaction between lipopolysaccharide (LPS) and TLR4 and between IL-1β and IL1R/IRAK1 [20], inducing translocation of the IKKβ/NF-kB complex to nucleus to promote NLRP3 and proIL-1β expression. While TLR4/Myd88 is required for an immediate early phase, the IL1R/IRAK1 axis is required for a subsequent intermediate phase of posttranslational NLRP3 activation [20], caspase-1 and pyroptosis and early IFN-gamma [21]. (2) Cytosolic deubiquitination of NLRP3 by TRAF6/IRAK/Myd88 TLR4 aggregates or by increased reactive oxygen species (ROS) and ATP concentrations to induce NLRP3 inflammasome assembly, relying on a “primed” cytosolic NLRP3. Both mechanisms are not mutually exclusive as bone marrow-derived macrophages (BMDMs) from NLRP3 knock-out (KO) did not activate apoptosis signals (expression of caspase 1) when stimulated with LPS and Nigericin, while the expression of caspase 1 was observed in wild-type mice and in NLRP3 KO in which NLRP3 was exogenously introduced [22]. In addition to these observations, it is to be acknowledged that all these mechanisms are further thought to involve multiple intracellular pathways including mitochondrial stress and lysosome activation. In fact, NLRP3-inflammasome activation induces increased K^+^ cellular efflux [23], which in turn stimulates cytosolic release of lysosomal cathepsins [24]. Cathepsins are then thought to favor 1) NLRP3 translocation into mitochondria [25,26,27] or 2) cytosolic release of mitochondria-derived factors such as ROS [28], oxidized mitochondrial DNA [29] and cardiolipin [30]. The second mechanism is specifically supported by the cardiolipin/NLRP3 axis, which is considered an important player of apoptotic signals. Physiologically, cardiolipin is present in the inner mitochondrial membrane to support enzymatic activities (like complex IV assembly) and to act as a proton trap to release or absorb protons excess during oxidative phosphorylation and maintain physiological pH. However, during increased cellular oxidative stress, lysosomial activation and NLRP3 assembly, it undergoes the activity of cardiolipin-specific oxygenases, which produce cardiolipin hydroperoxides translocating to the outer mitochondrial membrane inducing pores that facilitate the exit of apoptotic molecules like cytochrome c (cyt-c). Cyt-c interacts with inositol 1,4,5-trisphosphate (IP3) of the endoplasmic reticulum to induce calcium cytosolic release and induce further apoptotic signals. However, if NLRP3 switching is dependent on mitochondrial oxidative stress is debated. In some reports, pharmacologically blocking mitochondrial respiratory chain (in vitro by pyridaben) blunts the ROS intracellular levels, resulting in the inhibition of NLRP3 [31], non-transcriptional activation, whereas, in others, ROS production has been found to be essential for NLRP3 priming but not activation upon re-stimulation [22].

Therefore, despite mechanisms that have been hypothesized by robust data, a single unifying event has not emerged and there is no consensus on which is the first receptor system to trigger NLRP3 rapid activation. Moreover, these events do not occur all together to activate NLRP3 and further data support that some of them are associated with multiple inflammasome systems as well [32]. 

Multiple TLR systems have been demonstrated to be involved in NLRP3 assembly and apoptosis-induction, as apoptosis induced by LPS and Nigericin in BMDMs from NLRP3 KO mice primed with one-hour cycloheximide exposure was not marked by caspase 1 expression that was different from apoptosis induced by Flagellin, a TLR5 agonist [22]. However, caspase-1 was overexpressed in apoptotic BMDMs stimulated with cycloheximide, LPS and Nigericin when NLRP3 was exogenously expressed in mice genetically lacking NLRP3, even more than the activation with Flagellin [22], implying that NLRP3 assembly would be more sensitive to TLR4 activation.

Independent observations have then confirmed rapid LPS priming in NLRP3 KO BMDMs with NLRP3 expression reconstituted using a constitutive promoter, but have demonstrated that LPS potentiates AIM2 inflammasome activation as well to submaximal doses of cytosolic DNA without concomitant upregulation of AIM2 protein expression [33]. Therefore, the relevance of multiple receptor systems in rapid NLRP3 activation is not to be excluded.

In summary, TLRs systems can license very quickly NLRP3 or prepare a basal NLRP3 nuclear expression and synthesis; in this way a basal pool of pro-IL-1β and “primed” NLRP3 is ready to act after a second re-stimulation. This “transcriptional priming”, however, depends on the expression of other, multiple factors that need to be identified, and they cannot be unified in cellular oxidative stress as the common soil. After the second stimulation, multiple evidence supports that the “primed system” is able to rapidly release effectors of the innate immune response, that is IL-1β from its precursor form (pro-IL-1β) via NLRP3 assembly. In turn, circulating IL-1β may also promote the release of IL-6, another important player in the atherosclerotic process, by the liver [34].

On top of this evidence and beyond these molecular aspects calling for the need of an in-depth investigation, the scenario of a cellular priming, which is the prelude of a rapid exacerbated activation to sustain adequate response against pathogens, profoundly changed the previous notion of innate immune response. In fact, the term “trained immunity” has been recently coined by several authors starting from the observation in organisms lacking adaptive immunity that there was, in any case, resistance to reinfection [35].

## 4. Multiple Pathogenic Factors Converge to Promote ASCVD through Modulation of NLRP3 Inflammasome

Focal and systemic chronic low-grade inflammation through NLRP3 activation is a major player in ASCVD risk and disease, as discussed above. Interestingly, several environmental, genetic and epigenetic conditions are associated with ASCVD through the modulation of this pathway, often in combination. The main ones are discussed below.

### 4.1. Nutrition

One important environmental component is represented by unhealthy nutrition patterns. An unhealthy dietary intake has indeed been associated with increased risk of acute myocardial infarction globally, accounting for about 30% of the population-attributable risk [36]. Although the associated mechanisms leading to an inflammatory response promoting ASCVD risk are not fully unveiled, recent experimental and clinical evidence suggests the existence of a causal relationship between unhealthy nutrition profiles and NLRP3 inflammasome activation [37].

Moreover, the involvement of this specific pathway in diet-induced chronic diseases, like obesity, type 2 diabetes mellitus (T2DM) and metabolic syndrome, has also been highlighted [38] and is also related to the presence of dysfunctional visceral adipose tissue with altered adipokine and ghrelin patterns, resulting in turn with greater ASCVD risk [8,39,40,41,42]. Regular physical activity is a generally recommended companion of healthy nutrition. However, the effects of physical activity on the modulation of the inflammatory profile, with specific reference to the NLRP3 inflammasome, are not univocal in subjects with different training levels, suggesting an additional level of complexity in this response [43].

### 4.2. Microbiota

Closely related to the above-mentioned conditions, the gut microbiota is another pivotal player in this context, as substantial evidence is present that dysbiosis, i.e., imbalance in the gut microbiome composition and function, can influence ASCVD progression [44]. In addition to dysbiosis, alterations of intestinal barrier permeability results in direct contact of immune cells with antigens in the intestinal lumen, causing impairment of physiological barrier functions, such as the immune response to pathogens, and chronic inflammation, especially associated with NLRP3 inflammasome modulation [45,46,47,48]. In addition to dysbiosis, other factors can contribute to chronic inflammation, such as the so-called leaky gut barrier [49], and alterations of the gut-vascular barrier that might control the type of antigens that are translocated across blood endothelial cells, allowing to discriminate differently sized particles of the same nature [50]. 

Moreover, a relevant role of oral microbiota dysbiosis and periodontal bacteria has recently been demonstrated, since they are able to induce diseases and worsen the metabolic parameters of a cluster of chronic diseases, such as T2DM and CVD [51].

### 4.3. Cholesterol

It has been clearly shown that, in the atherosclerotic plaque, endocytosed LDL release cholesterol that upon crystallization may activate the NLRP3 inflammasome, resulting in the release of mature IL-1β and IL-18, associated with augmented atherosclerosis [52]. By contrast, the lack of caspase-1 exerts a protective effect against atherosclerotic lesion evolution, further implying the causative effect of inflammasome in atherosclerosis [53]. Cholesterol thereby represents one of the principal triggers of the inflammasome, and this is further supported by observations in which the lack of ATP-binding cassette (ABC) transporters in myeloid cells derived from LDL receptor (LDLR) KO mice stimulates the activation and cells proliferation, inducing their engulfment in the atherosclerotic plaque [53]. Recent data support that oxidized LDL may act as a first stimulus for NLRP3 activation. In fact, although the transition from a cholesterol-rich diet to a standard fat diet reduces systemic inflammation in LDLR KO mice, myeloid cells still preserve elevated capacity to respond to a subsequent immune stimulus [54], hypothesizing that NLRP3 per se might have role in the epigenetic reprogramming of myeloid cells. Moreover, in the presence of dysfunctional arterial intima, a wide set of additional factors and conditions, which are individually expressed, may actually contribute to systemic and focal low-grade chronic inflammation, thus promoting ASCVD progression. 

**Figure 2:**
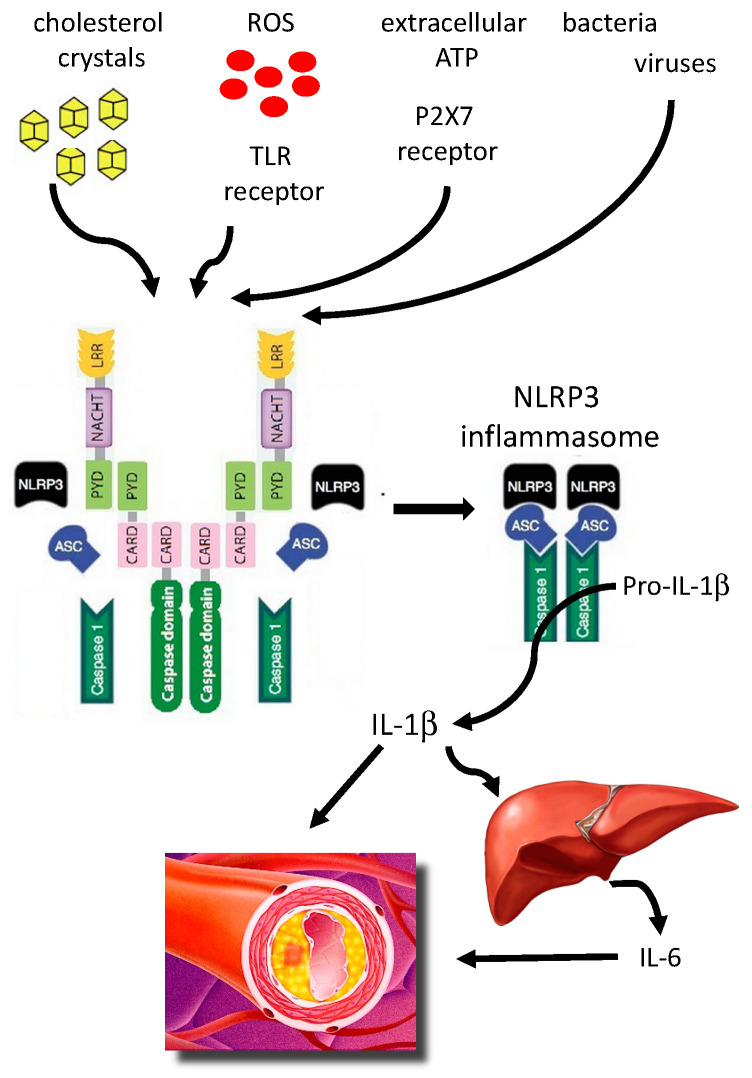
Different agents trigger (after a priming process) the activation of the NLRP3 inflammasome-IL-1β pathway to promote atherosclerosis. ASC, apoptosis-associated speck-like protein containing a caspase recruitment; ATP, adenosine triphosphate; CARD, caspase recruitment domain–containing protein; IL-1β, interleukin IL-1β; IL-6, interleukin 6; LRR, leucine-rich repeats; ROS, reactive oxygen radicals; TLR, Toll-like receptor.

Based on these observations, it thus appears that NLRP3 inflammasome activation is at the crossroads of several pathogenetic inputs promoting ASCVD (Figure 2). However, several of the above-described mechanisms, related to different aspects of NLRP3-associated low-grade inflammation, are not yet associated with specific diagnostic or predictive biomarkers, suggesting the need for identifying novel biomarkers and possibly treatment approaches. More specifically, we have a substantial lack of reliable biomarkers of NLRP3 activation targeted to the individual stratification of subjects to accurately determine ASCVD risk.

## 5. Role of NLRP3 Inflammasome Genetic Variants in ASCVD Pathophysiology

The currently available clinical biomarkers of inflammation, like high-sensitivity C-reactive protein (CRP), although useful in some conditions, are not specific indicators of NLRP3 inflammasome activation. Moreover, it is still unclear to what extent the mechanisms underlying the above-discussed concept of “trained immunity”, associated with NLRP3 inflammasome activation, may play a role in individual ASCVD pathophysiology. In addition, the quantification of circulating pro-inflammatory cytokines does not appear to be a robust approach in this field [55].

Interestingly, specific NLRP3-related gene variants have been found to be associated with a large set of autoinflammatory conditions, include psoriasis and rheumatoid arthritis [56], graft-versus-host disease [57], and macrovascular complications in type 2 diabetes mellitus [58], among others. Recent studies in mice and human patients unveiled several gain-of-function (GOF) mutations in inflammasome sensor proteins that allow inflammasome assembly in the absence of cognate ligands to trigger autoinflammatory syndromes. For example, cryopyrin-associated periodic syndromes (CAPS) are rare autoinflammatory diseases, comprising a broad disease spectrum with varying severity. CAPS are associated with GOF mutations in the NLRP3 inflammasome and the activation of IL-1β [59]. 

Several mutations in the NLRP3-inflammasome are associated with a high propensity for the development of several immune-mediated diseases. For example, in patients with CAPS, positional cloning revealed that heterozygous mutations in NLRP3 could be the cause of most cases with GOF mutations in NLRP3 gene located in chromosome 1q44 and associated with the constitutive activation of the NLRP3, leading to spontaneous activation of caspase-1 and an excess of IL-1β production [60]. Further, these mutations lead to the expression of constitutively active proteins able to induce NF-kB activation and IL-1β secretion dependent on ASC [61]. More than 90 different mutations in the NLRP3 gene have been identified in patients, showing a high prevalence of NLRP3 mutations in CAPS. However, only in a subset of known mutations of NLRP3 correlated with disease severity (Table 1) [62].

Therefore, a potential effective strategy in this direction may be the identification of variants of NLRP3-related genes associated with greater (or lower) ASCVD occurrence. Association studies have found that the Q705K polymorphism in the NLRP3 gene (rs35829419) conferred a protective effect against the risk of developing infarction in females and was also associated with increased C-reactive protein levels in males [63]. In another study, the single nucleotide polymorphism rs35829419 revealed a significant association with increased IL-1β levels, but showed a trend with lower levels of plasma CRP, suggesting a relevant complexity in the impact of these SNPs [64]. Regarding the variants of the CARD8 gene, the C10X variant (rs2043211) was found to be associated with lower expression of CARD8 in plaques as well as with a lower levels of CRP and monocyte chemoattractant protein-1 in plasma, but it showed no association with the risk of myocardial infarction in a Swedish cohort [65]. However, in a Chinese cohort, rs2043211 was associated with ischemic stroke [66]. A study on a Spanish cohort also showed that rs2043211 was not associated with the risk of developing cardiovascular events in RA patients [67]. Moreover, the interaction between the Q705K NLRP3 SNP and C10X SNP in the CARD8 gene (rs35829419/rs2043211, CC/AT versus CC/AA) conferred a modest protective effect against abdominal aortic aneurysms [64].

Another condition that may be explored in this context is heterozygous familial hypercholesterolemia, a relatively frequent condition (1:200–300), mostly showing inactivated low-density lipoprotein receptor (LDL-R) mutations and associated with elevated LDL-C and CV risk [68]. In these patients, the individual propensity to develop ASCVD may be further increased by the presence of selected SNPs associated with an (auto)pro-inflammatory NLRP3 pattern [69]. Collectively, taking these genetic findings into account, it is likely that these variants might modulate the basal active state of immune response and might thereby contribute to the pathophysiology of ASCVD or even reduce its prevalence in other cases. Data on genetic variations related to NLR3P inflammasome is still limited: larger and more robust studies are needed to clarify the relative contribution of each SNPs to CVD, taking also into consideration among others gender, country or ethnicity.

## 6. Identification of NLRP3-Related Gene Variants: A Useful Tool to Refine CVD Risk Assessment?

In light of the above-reported relevance of the activation of the NLRP3 inflammasome-IL-1β pathway for ASCVD progression and, in some cases, the apparently protective effects of some related SNPs, the assessment of a panel of NLRP3-associated SNPs may be reasonably implemented in the context of novel CVD risk algorithms which include genetic biomarkers. This may help to precisely define the individual autoinflammatory profile.

We recall that the current clinical approach to assess CVD risk in subjects in primary CVD prevention is based upon the use of relatively simple algorithms (Framingham Risk Score, Pooled Cohort Equations, QRISK2, and the Systemic Coronary Risk Evaluation (SCORE) system) relying on a small set of established phenotypical risk factors for CVD. Although useful for a general purpose, they actually show limited accuracy in predicting future CV events [70], resulting in substantial under/over-diagnosis and treatment for many people. Therefore, novel algorithms able to accurately predict such disease risk on an individual basis are urgently needed and should consider several important pathogenetic components, like genetic background, lifestyle aspects, comorbidities. Current research is indeed addressing this issue by integrating information from “omics” technologies with traditional risk factors and imaging data, using mendelian randomization studies [71,72] and artificial intelligence approaches [73,74]. In this context, a validated set of NLRP3-related SNPs may be added to a larger panel of relevant genomic information, to further refine the prediction of personal CVD risk. This strategy may especially help to identify subjects at a high/very high CV risk, therefore enabling the implementation of specific treatment strategies in the context of a precision medicine approach. 

Some issues clearly still remain to be assessed. For example, SNPs, including NLRP3-related ones, may individually have little effect on CVD risk, but, when present together, lead to a greater risk [75]. To evaluate the cumulative effect on CV risk, it is then important to combine these SNPs to generate genetic risk scores, useful to refine the prediction of an individual’s predisposition to overall CV risk, as they will also recapitulate the exposure time to risk factors. A clear understanding of which of these NLRP3-related SNPs are associated with the greatest impact on CV risk and how they do interact may offer a relevant benefit, as they will lead to effective therapeutic strategies for the early reduction of individual CV risk [76,77]. 

A validated CV risk stratification which includes also a NLRP3-related genomic score may then call for a specific pharmacological approach. Several agents have been shown to modulate this pathway and may be effectively developed for human use in the future. This group of agents includes inhibitors for the components of NLRP inflammasome (parthenolide, VX-740 and VX-765, Bay 11-7082, β-hydroxybutyrate, microRNA-223), indirect inhibitors (glyburide, JC124, FC11A-2), and direct inhibitors of NLRP3 protein (MCC950, 3,4-Methylenedioxy-b-nitrostyrene, CY-09, oridonin) [78,79]. Another potential approach is to inhibit mechanisms downstream of NLRP3 inflammasome activation, that is, for example, to interfere with IL-1β signaling by using anakinra, a recombinant IL-1 receptor antagonist that has anti-inflammatory and immunomodulatory actions, although no relevant effects in ASCVD prevention have been shown yet [80]. Interestingly, among drugs already in clinical use, statins, the most used drug class for hypercholesterolemia management, have been shown to modulate NLRP3 inflammasome, with both inhibitory and, in some cases, stimulatory effects [81].

In conclusion, the activation of NLRP3 inflammasome, triggered by several factors, including elevated cholesterol and genetic predisposition, has been clearly associated with ASCVD onset and progression. Therefore, this pathway should be taken into consideration at both the diagnostic and therapeutic levels in the context of the complex constellation represented by atherosclerosis.

## Figures and Tables

**Table 1:** Main gene variants (single nucleotide polymorphisms) related to the NLRP3-IL-1β pathway.

Gene	Single Nucleotide Polymorphism
NACHT leucine-rich repeat- and PYD-containing (NLRP)3 inflammasome	rs4353135, rs4266924, rs6672995, rs10733113, rs107635144, rs55646866, rs35829419
Caspase recruitment domain–containing protein (CARD)8	rs2043211, rs1972619
Caspase-1	rs501192, rs556205 and rs530537
IL-1 β	IL-1β (-511 and +3954)
IL-6	(-G174C); rs1800795)
